# Development and *in vitro* evaluation of a novel carboxymethyl chitosan–based potassium oxalate strip for dentinal tubule occlusion and penetration

**DOI:** 10.3389/fdmed.2026.1932702

**Published:** 2026-10-12

**Authors:** Monica Antony, Sruthy Prathap, Jayachandran Venkatesan, Vinita Boloor

**Affiliations:** 1 Department of Periodontology, Yenepoya Dental College, Yenepoya (Deemed to be University), Mangalore, India; 2 Yenepoya Research Centre, Yenepoya (Deemed to be University), Mangalore, India; 3 Centre for Nanomedicine, Directorate of Research, Malla Reddy Vishwavidyapeeth, Hyderabad, Telangana, India

**Keywords:** biocompatible material, carboxymethyl chitosan, dentin sensitivity, drug delivery system, potassium oxalate, scanning electron microscopy

## Abstract

**Introduction:**

Dentin hypersensitivity is characterized by a short, sharp pain arising from exposed dentin in response to external stimuli, as explained by the hydrodynamic theory. Although oxalate-based mouthrinses can reduce sensitivity by occluding dentinal tubules, their effectiveness may be limited due to brief contact time and rapid clearance by saliva.

**Objective:**

To develop and evaluate a novel potassium oxalate-incorporated carboxymethyl chitosan strip and compare its dentinal tubule occlusion, intratubular penetration and biocompatibility with a commercially available potassium oxalate mouthwash (Vantej) using an *in vitro* model.

**Methodology:**

A polymeric strip containing 1.6% potassium oxalate was fabricated using a carboxymethyl chitosan matrix. The strip was characterized using physicochemical properties including pH, viscosity, and thickness. Structural and morphological analyses were performed using Fourier Transform Infrared Spectroscopy and Scanning Electron Microscopy. Dentinal tubule occlusion and crystal deposition patterns were evaluated through SEM imaging. Cytocompatibility was assessed using a cell viability assay to determine the biocompatibility of the developed strip.

**Results:**

The fabricated strip demonstrated favourable physicochemical characteristics and successful incorporation of potassium oxalate. SEM analysis revealed dense and homogeneous crystal deposition on dentin surfaces treated with the strip. No statistically significant difference in occlusion scores was detected between the developed strip and the commercially available potassium oxalate mouthwash. Cell viability of exceeded 70%, indicating that the strip was non-toxic and biocompatible.

**Conclusion:**

The potassium oxalate-incorporated carboxymethyl chitosan strip exhibited favourable dentinal tubule occlusion, intratubular penetration and biocompatibility compared with the conventional mouthwash. The novel bioadhesive system may enhance the retention of desensitizing agents at dentin surfaces and provides a basis for further preclinical and clinical investigation for the management of dentin hypersensitivity.

## Introduction

Dentin hypersensitivity (DH) is a brief and intense pain arising from exposed dentine in response to external stimuli, such as thermal, tactile, evaporative, osmotic, or chemical triggers, which cannot be ascribed to any other dental defect or pathology (1). To have dentin hypersensitivity occur, there should be an exposure of the dentine (lesion localization) and open dentin tubules, which should communicate with the pulp (lesion initiation) (2). Some of the possible factors leading to DH are gingival recession, non-carious cervical lesions, abrasion, erosion, and periodontal disease (3). Stimuli to sensitivity include cold sensation, evaporative effect upon inhalation of air, brushing of teeth, acidic or sweet foods, all which affects the patients' quality of life negatively (4).

**Table 1:** Descriptive and inferential statistical findings for dentinal tubule occlusion and penetration depth.

Outcome	Potassium oxalate strip (*n* = 13)	Potassium oxalate mouthwash (*n* = 13)	Statistical test	Test statistic	*p*-value
Dentinal tubule occlusion score	Median = 3; IQR = 3.00; Range = 1–4	Median = 2; IQR = 2.00; Range = 1–3	Mann–Whitney U test	U = 54.0	0.109
Penetration depth (µm)	83.7 ± 45.3; Range = 16.1–142	84.1 ± 29.6; Range = 26.7–128	Independent-samples t-test	t = −0.026	0.979

**Table 2:** Cytocompatibility of the potassium oxalate-based strip assessed using the MTT assay.

Concentration (µg/mL)	Replicate 1 (%)	Replicate 2 (%)	Replicate 3 (%)	Mean ± SD (%)
10	101.4	105.9	109.3	105.54 ± 3.93
20	119.9	115.7	112.5	116.02 ± 3.70
30	122.7	120.7	125.1	122.85 ± 2.23
40	128.6	124.7	129.7	127.64 ± 2.64
50	138.1	134.3	132.8	135.06 ± 2.69
60	148.6	145.3	150.3	148.09 ± 2.51
70	153.4	157.6	155.1	155.38 ± 2.08
80	159.5	162.5	178.2	166.75 ± 10.05
90	161.0	162.6	166.0	163.16 ± 2.54
100	165.2	168.5	161.9	165.22 ± 3.32

The theory of hydrodynamics by Brännström is considered the most accepted hypothesis to explain the phenomenon. The theory states that any external stimulus generates fluid movement within open dentin tubules that trigger the response of mechanoreceptors innervated by A delta fibers from pulp (5). Hypersensitive dentin has been shown to exhibit a greater number of dentinal tubules with larger diameters compared to non-sensitive dentin (4). Consequently, current therapeutic approaches primarily focus on either occluding dentinal tubules to reduce fluid movement or suppressing neural transmission (5).

A wide range of desensitizing agents have been studied, including fluorides, oxalates, arginine, varnishes, adhesive resins, lasers, and desensitizing toothpastes or mouthrinses, for the management of DH (6). Of all the desensitisers, oxalate has attracted significant interest due to its capacity to create insoluble calcium oxalate crystals in dentinal tubules, decreasing dentinal permeability and fluid flow (7). In addition, the acid-resistant nature of calcium oxalate deposits contributes to sustained tubular occlusion (5). However, conventional formulations such as toothpastes and mouthrinses are limited by short intraoral contact time, rapid salivary clearance, and reduced localized delivery to sensitive sites (8).

To overcome these limitations, strip-based delivery systems have been recently introduced as targeted desensitizing modalities (9). These systems provide prolonged contact time, localized delivery, sustained release of active agents, and reduced salivary dilution, thereby enhancing tubular occlusion efficacy (10). Clinical and *in vitro* studies have demonstrated that oxalate-containing strips provide immediate and durable reduction in dentin hypersensitivity by promoting deep intratubular crystal deposition and reducing hydraulic conductance (9, 10). Hare et al. reported that oxalate strips achieved significantly greater dentinal fluid flow reduction compared with conventional desensitizing toothpastes and rinses (10), while Bahal et al. demonstrated superior clinical reduction in hypersensitivity scores with 1.6% potassium oxalate strips compared with arginine-calcium carbonate toothpaste (11). Similarly, Drake et al. observed that a single application of a 1.6% dipotassium oxalate strip provided effective reduction in hypersensitivity for up to four weeks (12).

Despite the promising performance of existing commercially available oxalate-based desensitising technologies, limited evidence exists regarding the development of a novel bioadhesive, polymer-based strip-based deliver system incorporating potassium oxalate. Carboxymethyl chitosan is a biocompatible, mucoadhesive and relatively inexpensive polymer that increases the localised retention of the desensitising agents while supporting scalable and potentially affordable therapeutic formulations. Thus, the current study aimed at formulating and evaluating a new carboxymethyl chitosan-potassium oxalate strip and comparing its physical properties, ability to occlude dentinal tubules, and penetration depth with that of an existing potassium oxalate mouth rinse.

## Materials and methods

This *in vitro* experimental study assessed the efficacy of the newly developed strips having potassium oxalate on the occlusion of the dentinal tubular orifices and depth of penetration, comparing it with the mouth wash containing potassium oxalate. This experiment has been carried out only after getting ethical approval.

The Inclusion criteria included teeth obtained from patients aged 18–35 years (to reduce variability in dentin sclerosis), human permanent premolars extracted for orthodontic or periodontal reasons, teeth with intact crowns and no visible cracks or fractures, teeth free from dental caries, restorations and cervical abrasion/erosion, teeth with fully formed roots and teeth without developmental defects such as enamel hypoplasia and dentinogenesis imperfecta. The exclusion criteria included teeth with caries, white spot lesions, restorations (composite, amalgam, GIC) and root caries, teeth with cracks, craze lines, and structural fractures, teeth with attrition, severe abrasion ans erosion defects, teeth with prior endodontic treatment and internal or external resorption, teeth with excessive calculus deposits affecting the dentin surface and teeth with developmental anomalies.

### Study design

The present *in vitro* experimental study was carried out after obtaining approval by the Scientific Review Committee and the Institutional Ethical Committee. Ethical approval for the study was obtained from the Institutional Ethics Committee (Approval/Reference No. YEC-1/2024/022). The extracted human teeth used in the study were handled in accordance with institutional procedures for human biological materials. Following completion of the experimental procedures, the specimens and associated biological waste were disposed of in accordance with the institutional biomedical waste management protocol.

### Methodology

#### Sample collection and group allocation

In total, 26 fresh extracted and anonymised human premolars were gathered from the Department of Oral and Maxillofacial Surgery at Yenepoya Dental College, Mangalore, after routine therapeutic extraction for orthodontic purposes

An *a priori* sample-size calculation was performed using G*Power software. The calculation was based on the reported mean flow reduction of 92.9% following treatment with an oxalate strip, as reported by Hare et al. (2016) (10), with an assumed effect size of 0.5. Based on these parameters, a total sample size of 26 specimens was determined, with 13 specimens allocated to each study group. The collected samples were divided randomly into two experimental groups (*n* = 13 per group):

- Group A: Treatment with 1.4% potassium oxalate (Vantej® mouthwash).
- Group B: Treatment with 1.6% potassium oxalate-based desensitizing strip (experimental formulation).

##### Synthesis of potassium oxalate-based desensitizing strip

The potassium oxalate-based hydrogel strip was synthesized by modifying the methodology described by Varoni et al. (2017) (13). Briefly, 2 g of sodium alginate was dissolved in 100 mL of distilled water and continuously stirred until a homogeneous gel was obtained. In a separate beaker, 3.2 g of potassium oxalate was dissolved in 100 mL of distilled water. Subsequently, 50 mL of the prepared sodium alginate solution was combined with 50 mL of the potassium oxalate solution, resulting in a final formulation volume of 100 mL containing 1.0 g of sodium alginate (1% w/v) and 1.6 g of potassium oxalate (1.6% w/v). PEG (10 mg) was incorporated as a plasticizer and 10 mg of carboxymethyl chitosan was incorporated as a mucoadhesive polymer. The resulting mixture was stirred until a homogeneous biocomposite solution was obtained. Approximately 10 mL of the prepared solution was transferred to a sterile glass Petri dish and dried at 50 °C for 12 h in a hot-air oven for solvent removal and film formation. The dried film was subsequently removed and cut into standardized strips using a sterile scalpel blade. Finally, the dried film was separated and cut into standard strips by using sterilized blade of scalpel as shown in Figure 1.

**Figure 1:**
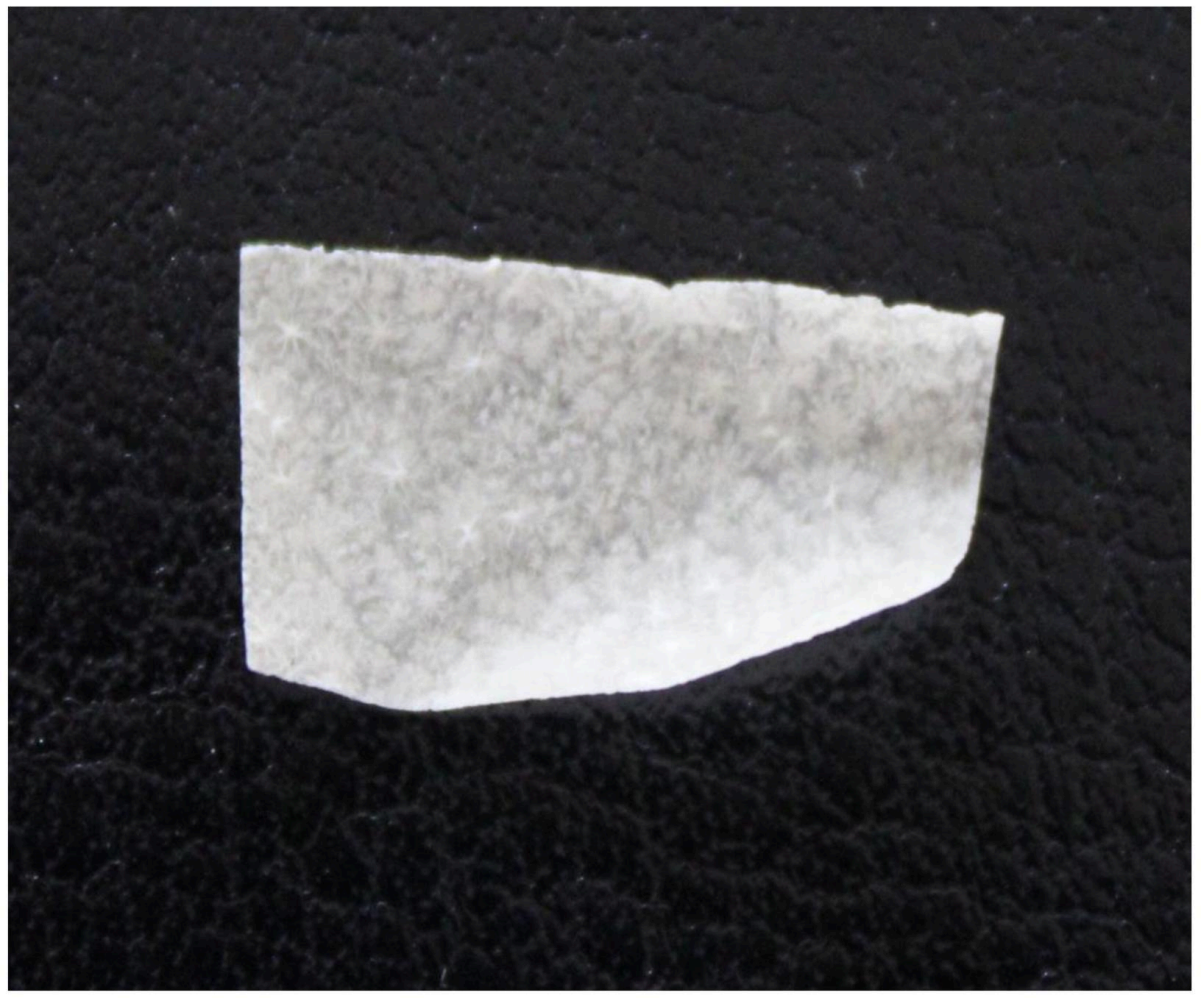
Potassium oxalate-based strip after drying.

##### Preparation of dentin discs

Decoronation of each tooth was done using a diamond disc at the level of CEJ with continuous flow of water. Root parts were then discarded. Perpendicular cuts were performed on each tooth crown to obtain a flat surface made of dentin parallel to the long axis of the tooth. A second cut about 3 mm away from the previously created dentin surface produced dentin discs with a dimension of 3 mm thick. Each specimen was then marked on the pulpal side, and its exposed coronal dentin surface was polished using 600-grit silicon carbide paper. Hypersensitive dentin with open dentinal tubules was simulated and smear plugs were removed by treating all specimens with 17% EDTA for 5 min. Specimens were then washed thoroughly using distilled water and allowed to air dry. Following preparation of specimens, they were all incubated in artificial saliva kept at room temperature.

### Treatment protocol

#### Group A – mouthwash treatment

Specimens in Group A were immersed and gently agitated in 1.4% potassium oxalate mouthwash for 1 min, twice daily, for 28 days.

#### Group B – desensitizing strip treatment

Specimens in Group B were treated with the 1.6% potassium oxalate-based strip. A single strip was applied once to the designated dentinal surface. The strip was designed as a single-use formulation that dissolved upon contact with saliva; therefore, no repeated strip applications were performed during the experimental period.

### Characterisation of the potassium oxalate gel

#### Fourier transform infrared spectroscopy (FTIR)

The FTIR helped to determine the presence of functional groups to characterise the strip which comprises of potassium oxalate, polyethylene glycol, carboxymethyl chitosan, and sodium alginate. Additionally, it was employed to identify the appearance or disappearance of peaks, which could be a result of molecular interaction or incompatibility of the strip composition. Spectroscopic analysis was carried out using a Bruker Alpha FT-IR Spectrophotometer (Bruker Optics GmbH, Ettlingen, Germany) with Attenuated Total Reflectance (ATR) accessory. The spectrophotometer operated on the OPUS 7.5 software (Bruker Optics GmbH, Ettlingen, Germany). Spectra were taken in the range of 4,000–400 cm⁻^1^ with a resolution of 4 cm⁻^1^ for each measurement. Characteristic peaks correspond to the functional groups of the formulation components, including:

- O–H stretching vibrations (carboxymethyl chitosan and alginate)
- C = O stretching of carboxylate groups (oxalate and alginate)
- C–O–C stretching vibrations (polyethylene glycol)
- Amide-related peaks (chitosan derivative)

The absorption bands observed in the FTIR spectrum of the final strip formulation were assigned by comparison with characteristic wavenumber reported in the literature for the individual constituent materials, including sodium alginate, polyethylene glycol (PEG), potassium oxalate/oxalate ions, and carboxymethyl chitosan. Individual FTIR spectra of these components were not obtained separately in the present study. Absence of significant peak displacement or disappearance was interpreted as compatibility between potassium oxalate and the polymeric excipients. FTIR analysis was carried out at NITTE, NGSM Institute of Pharmaceutical Sciences, Paneer, Deralakatte, Mangaluru.

### Scanning electron microscope (SEM) with energy dispersive x-Ray (EDS)

#### Sample preparation for SEM

Following treatment with the respective desensitizing agents, the dried specimens were mounted on for sputter coating, using a JEOL JFC-1,600 Auto Fine Coater (JEOL Ltd., Tokyo, Japan). A gold layer of approximately 25 nm thickness was deposited under vacuum conditions. SEM analysis was performed using a JEOL JSM-IT300 Scanning Electron Microscope (JEOL Ltd., Tokyo, Japan) operating at an accelerating voltage of 5 kV. Micrographs were obtained at standardized magnifications (×500 or higher). Image acquisition and analysis were carried out using JEOL SEM Control Software (JEOL Ltd., Tokyo, Japan) and further quantified using ImageJ software (National Institutes of Health, USA) for tubule counting and measurement. The SEM and EDS analyses were carried out at Nano Watts Technologies Pvt. Ltd., Bengaluru.

SEM images were evaluated by a single rater at the SEM analysis facility using the predefined criteria for assessment of dentinal tubule occlusion and penetration. Prior to SEM analysis, the samples were coded, and the rater was unaware of the identity of the coded samples and their corresponding treatment groups (potassium oxalate strip or mouthwash). Thus, SEM assessment was performed under blinded conditions with respect to treatment-group allocation.

#### Evaluation of dentinal tubule occlusion

Tubular occlusion was assessed following protocols described in dentin hypersensitivity SEM studies (Greenhill and Pashley, 1981). From each specimen, photomicrographs were captured at ×1,500 magnification from standardized central regions of the treated dentin surface. For each photomicrograph:

- Total number of visible dentinal tubules were counted.
- Number of completely occluded tubules were counted.

Based on the level of the occlusion, the specimens were scored from 1 to 4 (14):

1. No Occlusion- Majority of dentin tubules are open; tubule lumens clearly visible; no deposits observed (approx. 100% of tubules are unsealed)
2. Partial Occlusion- Some tubules partially blocked; tubule openings still visible; irregular or discontinuous deposits (approx. 25% to <50% of tubules are sealed)
3. Substantial Occlusion- Majority of tubules are occluded or narrowed; few open tubules remain (approx. 50% to <100% of tubules are sealed)
4. Complete/Near-Complete Occlusion- Tubules fully or almost fully occluded; continuous surface coverage observed (approx. 100% of tubules are sealed)

#### Evaluation of depth of penetration

To assess intratubular penetration depth of the desensitizing material, treated specimens were sectioned longitudinally using a low-speed diamond disc and the cross-sectioned specimens were dehydrated and sputter-coated as described previously. SEM images were captured of the cross-sectional view of the tooth at ×500 and ×1,000 magnification to visualise intratubular precipitate extension. Penetration depth was measured from the dentin surface of randomly selected tubules per specimen, to the deepest visible extent of intratubular occluding material using ImageJ software (NIH, USA) calibrated against the SEM scale bar (15).

### P^H^

The procedure for determining the pH involved taking an accurate weight of 1 gram of the fabricated strip and dissolving it in 25 mL of distilled water in the presence of magnetic stirring. The measurement was taken using a pH meter (µ pH System 361, Systronics India Ltd., Ahmedabad, Gujarat, India), at the Yenepoya Research Centre. The device was calibrated before measuring the pH value using pH buffers of 4.0, 7.0, and 9.2, following the instructions provided by the manufacture (16).

### Viscosity

The rheological behavior of the newly designed potassium oxalate incorporated desensitizing strip formulation was evaluated to determine its flow characteristics and viscosity profile under controlled shear conditions. Rheological measurements were performed using a controlled-stress rotational rheometer (Anton Paar MCR 92, Anton Paar GmbH, Graz, Austria) at the Yenepoya Research Centre. Approximately 1 g of the prepared formulation was dissolved in 25 mL of distilled water and carefully placed on the lower measuring plate of the rheometer. A flow curve analysis was performed by subjecting the formulation to a controlled shear rate sweep ranging from 0.1 s⁻^1^ to 100 s⁻^1^. Shear stress (*τ*) and viscosity (*η*) values were plotted against shear rate (γ˙). The rheological parameters yield stress (*τ*₀), flow consistency index (k), and flow behavior index (n) were calculated by fitting the experimental results into the Herschel-Bulkley equation.

### Thickness

The thickness of the potassium oxalate–incorporated desensitizing strip was determined using a Digital Vernier Calliper (Model: 1,108–150, Aerospace™, India) with a least count of 0.01 mm as shown in Figure 2. Ten strips were randomly selected from each of the batches and the thickness was measured. Measurement was performed at Yenepoya Research Centre (16).

**Figure 2:**
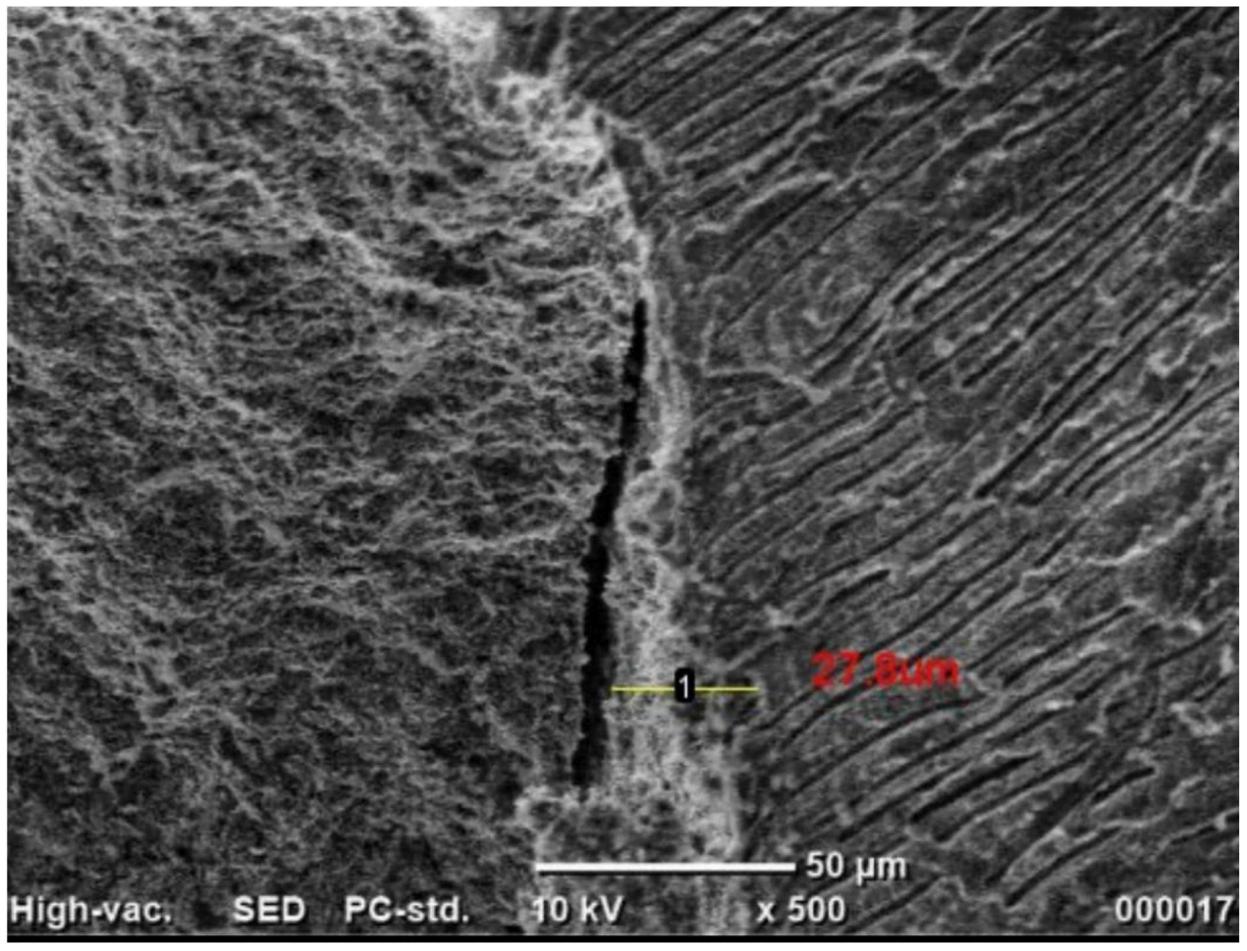
SEM of tabular occlusion of dentin disc treated with potassium oxalate-based mouthwash.

### Biocompatibility

The *in vitro* cytocompatibility of the synthesized carboxymethyl chitosan-based potassium oxalate strip was evaluated using the MTT colorimetric assay. The strip extract was tested at concentrations of 10, 20, 30, 40, 50, 60, 70, 80, 90, and 100 µg/mL. Untreated cells served as the control and were normalised to 100% cell viability. The MTT assay was independently repeated three times (*n* = 3). Cell viability was calculated relative to the untreated control and expressed as a percentage. The results were presented as mean ± standard deviation (SD).

#### Cell line and culture conditions

Mouse fibroblast cell line L929 (NCCS, Pune, India), widely recommended for cytotoxicity testing under ISO standards, was used for the assay. Cells were cultured in Roswell Park Memorial Institute (RPMI-1640) medium supplemented with 10% fetal bovine serum (FBS), 1% penicillin–streptomycin solution. A pre-culture of L929 cells was performed by seeding approximately 1 × 10⁴ cells per well into sterile 96 well tissue culture plates. These plates were incubated for 24 h at 37 °C with 5% CO₂ to allow for cell attachment and monolayer formation.

#### MTT assay procedure

After the completion of culture incubation, the supernatant fluid of each cell sample was aspirated carefully. MTT solution in a concentration of 0.5 mg per mL in PBS was then added to each of the cell wells in 100 µL volumes. The cells were then incubated for four hours at 37 degrees Centigrade in an environment of 5% CO₂. In this experiment, the live cells having active mitochondrial dehydrogenase converted the yellow MTT tetrazolium salt to insoluble purple formazan crystals.

Post-incubation procedure:

- The MTT solution was removed.
- 100 µL of dimethyl sulfoxide (DMSO) was added to each well to solubilise the formed formazan crystals.
- The plate was gently agitated and incubated for 15 min at 37 °C to ensure complete dissolution.

#### Optical density measurement

The absorbance was measured at 595 nm using the instrument iMark™ MicroplateReader by manufacturer Bio-Rad Laboratories Inc., Hercules, California, USA using the Microplate Manager® Software (Bio-Rad Laboratories, USA). All biocompatibility experiments were carried out at: Yenepoya Research Centre, Yenepoya (Deemed to be University), Mangaluru, Karnataka, India.

Percentage of cell viability was calculated as follows:

$$Cell\;Viability\;(\text{\%})=\frac{OD_{sample}}{OD_{control}}\times 100$$

According to ISO 10993-5, a cell viability greater than 70% was considered non-cytotoxic (17).

## Results

On synthesis of the potassium oxalate-based gel, it was subjected for the characterisation tests:

### Evaluation of pH

The pH of the synthesized potassium oxalate–incorporated gel for the strip was found to be 6.74. The analysis was performed in triplicate, and consistent readings were obtained across all measurements. No abrupt acidic or alkaline variations were observed during the evaluation.

### Evaluation of viscosity

The viscosity started from 670.5 mPa.s at the first recorded shear rate and ended at 14.34 mPa.s at the last recorded shear rate. The interpolation of shear rate 50 s-1 showed that the viscosity is equal to 14.116 mPa.s.

The rheology of the novel strip formulation was also evaluated using the Herschel-Bulkley model and was plotted as the shear stress-shear rate curve. The results obtained from the experiments were modelled using the Herschel-Bulkley equation, resulting in the following parameters:

- Yield stress (*τ*₀): 0.04064 Pa
- Consistency index (b): 0.024664
- Flow behaviour index (p): 0.8579

Flow behavior was found to show an increase in shear stress with an increase in shear rate. The value of the flow behavior index (p) was found to be less than 1. There was some yield stress observed before the initiation of flow for this formulation.

### Evaluation of thickness

Ten measurements of the developed potassium oxalate strip yielded a mean thickness of 0.0243 ± 0.0010 mm, with individual measurements ranging from 0.023 to 0.026 mm.

### Evaluation of FTIR

To develop the potassium oxalate-based strip, alginate–potassium oxalate–PEG–carboxymethyl chitosan was used, hence complex FT-IR spectrum was detected. The FTIR peak assignments represent the correlation between observed vibrational frequencies and the corresponding functional groups of the polymeric components and potassium oxalate, confirming successful incorporation and chemical compatibility within the developed strip formulation as represented in the graph Figure 3.

**Figure 3:**
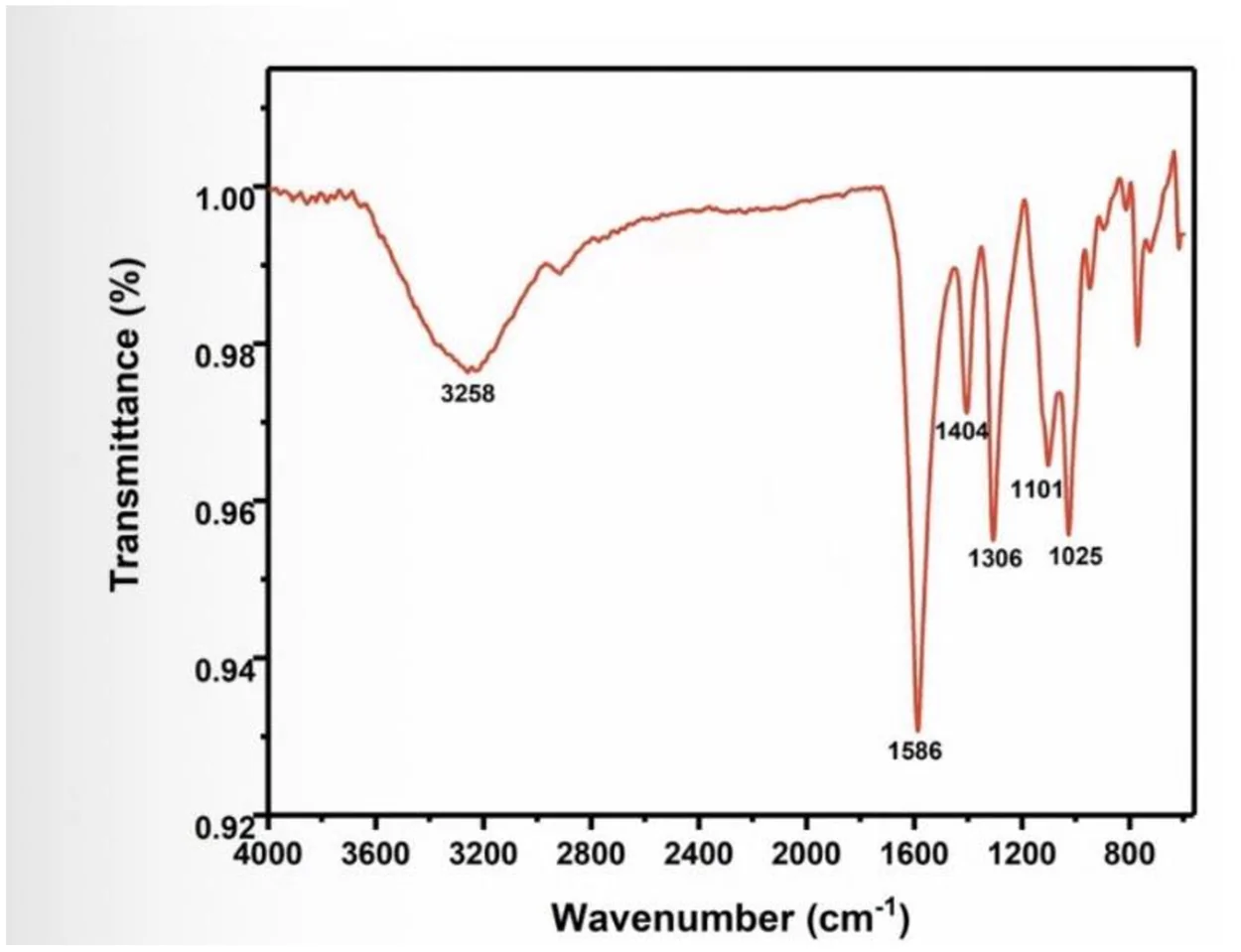
FTIR analysis of the developed potassium oxalate incorporated carboxymethyl chitosan strip.

FTIR peak assignments of the developed potassium oxalate strip based on literature-reported characteristic wavenumbers of its constituent materials as follows:

- 3258 cm⁻^1^ – Broad O–H stretching vibration; Component Attribution: Alginate, PEG, Carboxymethyl chitosan; Interpretation: Indicates presence of hydroxyl groups; confirms hydrophilic nature and hydrogen bonding capability of polymer matrix.
- 1586 cm⁻^1^ – Asymmetric stretching of –COO⁻ groups; Component Attribution: Alginate, Carboxymethyl chitosan, Potassium oxalate; Interpretation: Confirms presence of carboxylate groups and oxalate ions within formulation.
- 1404 cm⁻^1^ – Symmetric stretching of –COO⁻ groups; Component Attribution: Alginate, Carboxymethyl chitosan, Potassium oxalate; Interpretation: Supports integrity of polymer backbone and oxalate incorporation.
- 1306 cm⁻^1^ – Symmetric O–C–O stretching vibration; Component Attribution: Potassium oxalate; Interpretation: Characteristic oxalate ion vibration indicating successful active ingredient incorporation.
- 1101 cm⁻^1^ – C–O–C stretching vibration (glycosidic linkages); Component Attribution: PEG, Alginate, Carboxymethyl chitosan; Interpretation: Confirms presence of polysaccharide backbone and PEG structure.
- 1025 cm⁻^1^ – C–O stretching vibration; Component Attribution: PEG, Alginate, Carboxymethyl chitosan; Interpretation: Characteristic polysaccharide ether linkage; supports structural integrity of polymer matrix.

### Evaluation of SEM

Scanning Electron Microscopy (SEM) was employed to assess the dentinal tubule occlusion and penetration depth following application of the potassium oxalate strip and potassium oxalate-based mouthwash on dentin discs. A total of 13 samples were analysed in each group.

#### Dentinal tubule occlusion

The dentinal tubules were examined for their occlusion level according to a scoring scale, and the mean occlusion score was obtained for each group as the sum of all scores divided by the number of samples per group. This average value represents the overall occlusion performance of each treatment group. The strip treated group demonstrated a higher average occlusion score (2.69) as shown in Figure 4, compared to the mouthwash-treated group (2.00) as shown in Figure 5.

**Figure 4:**
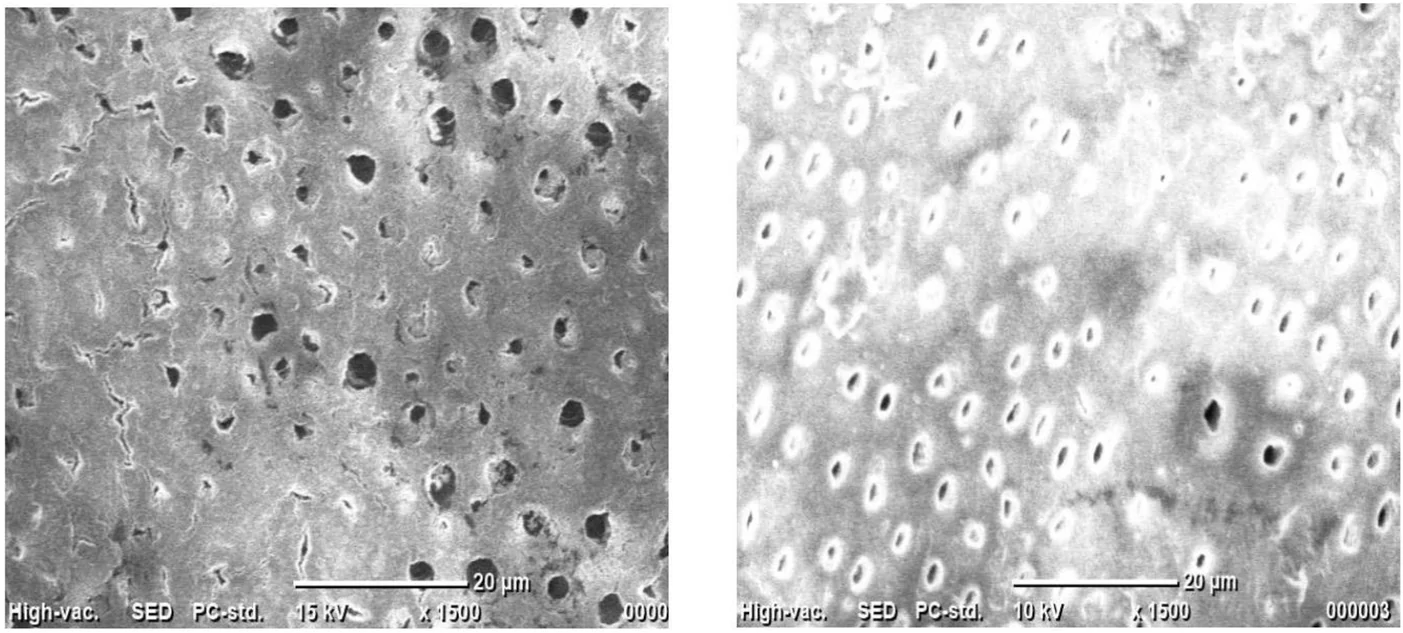
SEM of tubular occlusion of dentin disc treated with potassium oxalate-based strip on the left and right potassium oxalate-based mouthwash on the right.

**Figure 5:**
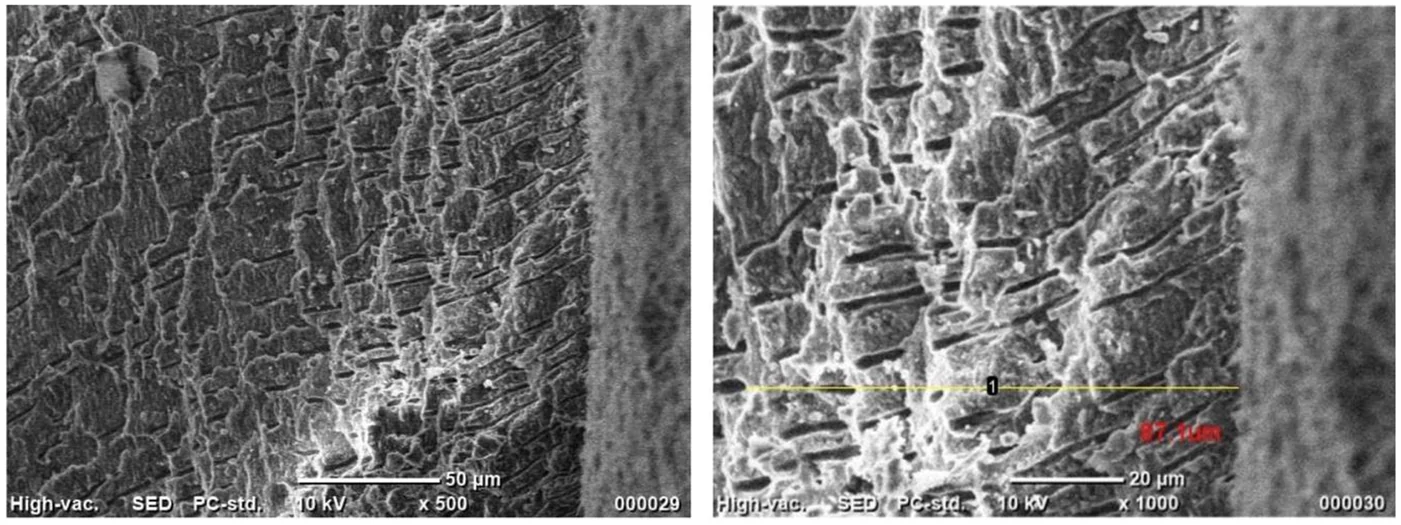
SEM of tabular penetration of dentin disc treated with potassium oxalate-based strip.

For statistical analysis, occlusion being an ordinal variable was summarised using median and interquartile range (IQR). The strip group showed a median occlusion score of 3 (IQR: 3.00; range: 1–4), whereas the mouthwash group showed a median score of 2 (IQR: 2.00; range: 1−3). For the intergroup comparison, the Mann–Whitney U test was applied, resulting in U = 54.0 and *p* = 0.109. This difference between the groups were not statistically significant (*p* > 0.05). The distribution of individual occlusion scores for both groups is presented in Figure 6.

**Figure 6:**
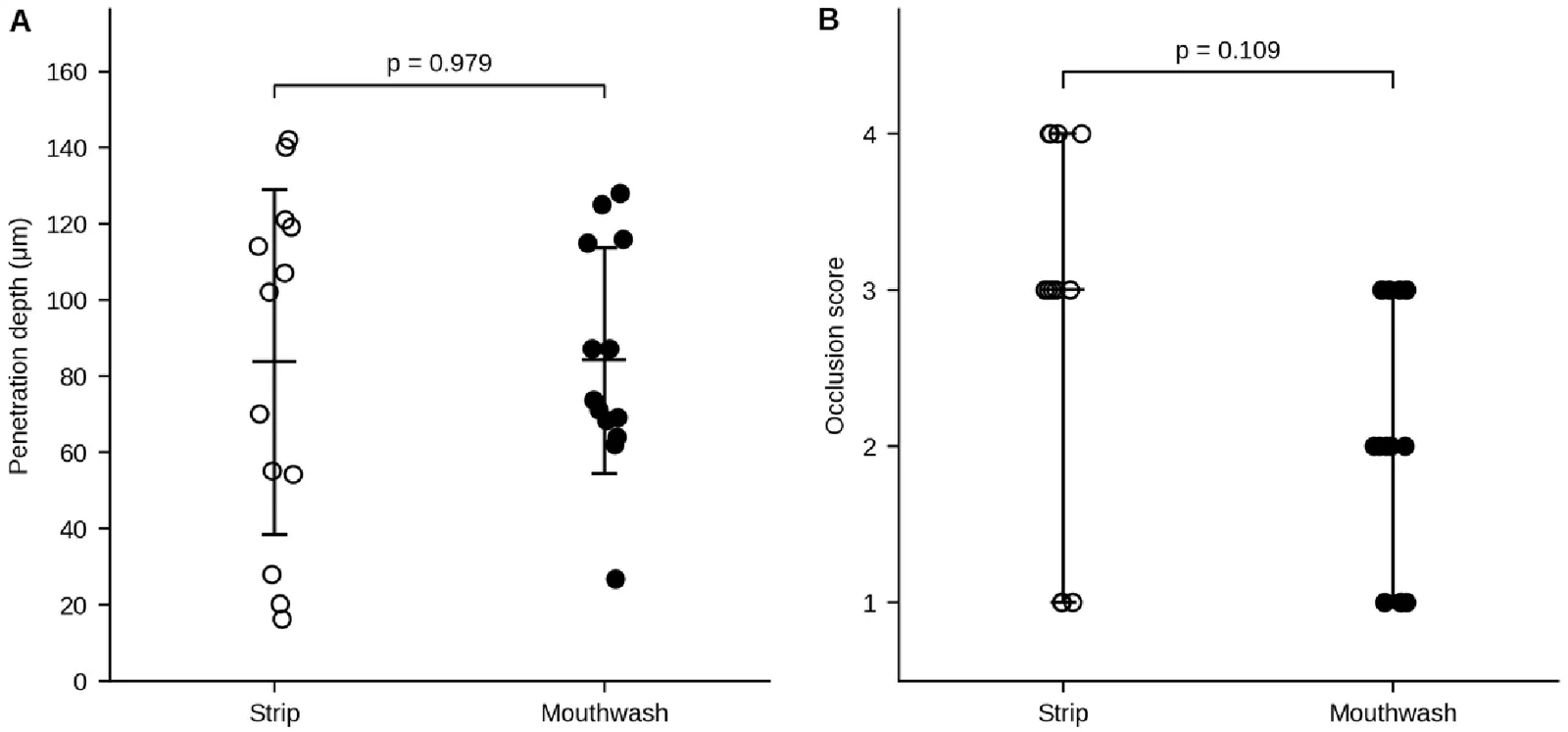
Occlusion dot plot and penetration-depth dot plot.

#### Penetration depth

The maximum penetration depth observed in the strip group was 142 µm, whereas the mouthwash group exhibited a maximum penetration depth of 128 µm. The strip group demonstrated a mean penetration depth of 83.7 ± 45.3 µm (range: 16.1–142 µm), while the mouthwash group demonstrated a mean of 84.1 ± 29.6 µm (range: 26.7–128 µm) as shown in Figure 5. The corresponding cross-sectional SEM image of the mouthwash-treated group is shown in Figure 2, and the distribution of individual penetration-depth values for both groups is presented in Figure 6.

Normality testing using the Shapiro–Wilk test indicated normal distribution of the data in both groups (Strip: W = 0.906, *p* = 0.162; Mouthwash: W = 0.923, *p* = 0.277). Therefore, Student's independent samples t-test was performed for intergroup comparison. The t-value was −0.026 with a *p*-value of 0.979, indicating no statistical significance (*p* > 0.05).

### Evaluation of biocompatibility

Morphological assessment of the strip extract was performed under 4X and 10X magnifications.

At 4X magnification (before treatment), L929 cells exhibited normal fibroblastic morphology with uniform distribution and formation of a confluent monolayer. The cells appeared elongated and spindle-shaped, with intact cellular architecture. At 10X magnification (before treatment), the cells demonstrated well-defined cytoplasmic extensions and maintained cell-to-cell contact, consistent with healthy fibroblast morphology. No signs of cellular degeneration were observed.

Following exposure to the strip extract:

At 4X magnification (after treatment), the overall cell density and distribution remained comparable to the pre-treatment condition. The monolayer integrity was preserved, and no noticeable areas of cell detachment were observed. At 10X magnification (after treatment), treated cells retained their characteristic spindle-shaped morphology and adherence to the culture surface. There was no evidence of cytotoxic changes such as cell rounding, shrinkage, membrane blebbing, or cellular debris.

#### Quantitative MTT assay results

The MTT assay demonstrated that cell viability remained above the 70% threshold, specified by ISO 10993-5 for classification as non-cytotoxic. The absorbance values indicated maintained mitochondrial metabolic activity following exposure to the strip extract. Both morphological observations and quantitative MTT results indicate that the potassium oxalate–based strip exhibits no cytotoxic effects on L929 fibroblast cells under the tested conditions and can be considered biocompatible *in vitro*.

## Discussion

SEM examination demonstrated dentinal tubule occlusion following treatment with the developed potassium oxalate strip. The strip group showed a median occlusion score of 3, whereas the mouthwash group showed a median score of 2. However, the intergroup difference was not statistically significant (Mann–Whitney U = 54.0, *p* = 0.109). These findings indicate that tubule occlusion occurred following application of the developed strip under the experimental conditions employed. Importantly, the lack of statistical significance should not be interpreted as evidence that the two formulations are equivalent or have comparable efficacy, since the present study was not designed as an equivalence or non-inferiority investigation.

Potassium oxalate acts by the precipitation of calcium oxalate crystals resulting from the interaction between the calcium ions in the dentinal fluid and potassium oxalate (7, 18). The numerically higher occlusion score observed in the strip group may be related to the extended period of contact facilitated by the polymeric matrix, although this difference did not reach statistical significance and should be interpreted with caution. Sustained availability of oxalate ions increases the probability of intratubular crystal nucleation and growth prior to salivary clearance. Greenhill and Pashley (1981) (18) demonstrated that oxalate treatment significantly reduces dentinal permeability through intratubular precipitation. Suge et al. (1995) (19) reported that oxalate application produces calcium oxalate crystals that effectively occlude tubules under SEM evaluation. Wang et al. (2011) (15) in their SEM evaluation of desensitizing materials, noted that materials providing sustained contact exhibited more complete tubule occlusion compared to transient applications. Similarly, Varoni et al. (2017) (13) demonstrated enhanced occlusion with hydrogel-based oxalate systems due to increased residence time at the dentin interface. The present findings are consistent with these studies, particularly in showing tubular occlusion.

Interpretation of tubular penetration shows that the values of the mean penetration depth were comparable between the strip (83.7 ± 45.3 µm) and mouthwash (84.1 ± 29.6 µm) groups. This suggests that both the delivery systems allowed oxalate ions to diffuse into dentinal tubules to similar average depths. Although the maximum observed penetration depth was 142 µm in the strip group and 128 µm in the mouthwash group, maximum individual values should not be used to infer superior penetration by either formulation. The considerable variability in penetration depth, particularly in the strip group, may reflect heterogeneity in dentinal tubule characteristics and deposition within the examined specimens. Thus, the findings demonstrate that intratubular penetration occurred with both treatment approaches under the conditions evaluated, without establishing superiority or equivalence between them. The importance of penetration depth lies in the higher level of protection from mechanical abrasion and acidic environment when crystals are deposited deeper (20). Suge et al. (19) found that crystals formed deeper in the tubules have greater acid resistance than surface-formed ones.

The pseudoplastic behavior with a finite yield stress value provides an example of adaptation and retention. It has been reported that mucoadhesive polymers tend to prolong the duration and local accumulation of drugs relative to those from liquid formulations (21). Even though mouthwashes successfully release oxalate, the therapeutic effect relies upon multiple applications to sustain tubule blockage (22). The developed strip represents a localised delivery system designed to overcome the limitations of conventional mouthwashes. The localised delivery system enhances retention at the target site, and such characteristics could help the future development of accessible and patient-friendly desensitizing therapies, particularly in settings where sustained local delivery systems are limited. The shear-thinning behaviour in the present study provides additional information regarding the physical characteristics of the developed formulation. The reduction in viscosity with increasing shear rate indicates non-Newtonian flow behaviour, which may facilitate spreading and adaptation of the formulation during application. The Herschel–Bulkley flow behaviour index was below 1, further supporting the shear-thinning nature of the formulation. Nevertheless, these rheological findings describe the flow characteristics of the formulation and should not be interpreted as direct evidence of prolonged retention or controlled drug release, as these properties were not specifically evaluated in the present study.

The near-neutral pH (6.74) is within the physiologically acceptable range for oral tissues and is higher than the critical pH threshold for demineralisation of dentin (23). The near-neutral pH observed for the developed formulation is relevant because the physicochemical environment at the dentin surface may influence both the stability of the substrate and mineral precipitation. In the context of the present study, the measured pH demonstrates that incorporation of potassium oxalate into the polymeric matrix resulted in a formulation with a near-neutral pH upon dispersion. In comparison, although commercial mouthwashes are also formulated within acceptable pH ranges, they do not maintain a localized pH environment at the dentin interface due to rapid dilution.

The consistent uniform thickness of the strip provides reliable loading of the drug, flexibility, and close adaptation to dentinal surfaces. Oral dissolving films in the range of 20–100 µm have been reported to provide optimal balance between mechanical strength and patient comfort (16). The relatively narrow variation in strip thickness further indicates consistency among the measurements obtained from the prepared formulation. Uniform thickness is an important physical characteristic of film-based formulations because substantial variations could influence handling and the amount of formulation present within a given strip area.

Chitosan and alginate-based systems are well documented for their favourable biological profiles (24). The FTIR spectrum of the developed strip exhibited characteristic absorption bands, which were assigned by comparison with literature-reported FTIR characteristics of the constituent materials. While mouthwash formulations are generally safe due to brief exposure, the strip demonstrates safety even under sustained contact conditions, which is essential for a delivery system designed to remain *in situ* during dissolution.

The MTT assay provided additional information regarding the *in-vitro* cytocompatibility of the developed formulation. Cell viability remained above 70% across all tested concentrations, indicating that no cytotoxic effect was observed under the experimental conditions employed. The quantitative findings were supported by morphological observations of the L929 fibroblast cells, which retained their characteristic spindle-shaped appearance without evident cytotoxic morphological alterations. These findings support the cytocompatibility of the formulation under the conditions tested; however, an *in-vitro* MTT assay alone cannot establish clinical biological safety.

Recent evidence continues to support dentinal tubule occlusion as an important therapeutic approach for the management of dentin hypersensitivity. Contemporary reviews have highlighted the development of materials and techniques aimed at achieving effective tubule occlusion, with increasing emphasis on the depth, stability, and durability of the occluding deposits (25). Systematic reviews have also demonstrated that different tubule-occluding materials can reduce dentin hypersensitivity, although variations in formulation, application protocols, and study methodology influence treatment outcomes (26). Furthermore, recent clinical evidence indicates that desensitizing agents acting through tubular occlusion can provide sustained reduction in dentin hypersensitivity, while emphasising the need for well-designed long-term studies to establish the relative effectiveness of different approaches (27). These findings support continued investigation of localized formulations designed to promote dentinal tubule occlusion; however, the clinical effectiveness and durability of the developed potassium oxalate strip require confirmation through appropriately designed *in-vivo* and clinical studies (28).

The present study has some limitations. Drug-release kinetics of potassium oxalate from the developed strip were not evaluated, limiting interpretation of the time-dependent release behaviour of the formulation. The present investigation was conducted under *in-vitro* conditions, and therefore the findings may not fully reflect the influence of the oral environment, including salivary flow, pH variations, mechanical forces, and other intraoral factors. Future studies should incorporate drug-release kinetics, and subsequent *in-vivo* or clinical evaluation (29).

## Conclusion

The carboxymethyl chitosan–potassium oxalate strip showed favourable physicochemical characteristics, successful incorporation of potassium oxalate, acceptable biocompatibility, and dentinal tubule occlusion characteristics comparable to those of a commercially available potassium oxalate mouthwash. The bioadhesive strip platform may enhance localized delivery and retention of desensitizing agents and represents a promising candidate for further preclinical and clinical evaluation in the management of dentin hypersensitivity (Supplemenatry Material).

## Data Availability

The original contributions presented in the study are included in the article/Supplementary Material, further inquiries can be directed to the corresponding author
