# Supplementary material for: Development and in vitro evaluation of a novel carboxymethyl chitosan–based potassium oxalate strip for dentinal tubule occlusion and penetration
Source: Front Dent Med. 2026 Oct 12;7:1932702. doi: 10.3389/fdmed.2026.1932702 (PMC13726520; doi:10.3389/fdmed.2026.1932702)
Supplement: Supplementary file 1 [file Table1.docx]

**Figures:**

**Figure 1: potassium oxalate strip preparation**

**Figure 2 measuring thickness using digital vernier calliper**

**Figure 3: pH meter**

**Figure 4: pH meter**

**Figure 5: SEM armamentarium**

**Evaluation of biocompatibility**

 **Figure 6: Photomicrograph of L929 fibroblast cells at 4X magnification before treatment**

.

**Figure 7: Photomicrograph of L929 fibroblast cells at 10X magnification before treatment**

**Figure 8: Photomicrograph of L929 fibroblast cells at 4X magnification after treatment**

**Figure 9: Photomicrograph of L929 fibroblast cells at 10X magnification after treatment**

**Graphs**

**Graph 1: Viscosity profile of the developed desensitizing strip formulation illustrating shear-thinning behavior as a function of shear rate**

**Graph 2: Flow curve showing the relationship between shear stress and shear rate of the developed desensitizing strip formulation fitted to the Herschel–Bulkley model**

**Tables:**

| Wavenumber (cm⁻¹) | Assigned Functional Group / Vibrational Mode | Component Attribution | Interpretation |
| --- | --- | --- | --- |
| 3258 cm⁻¹ | Broad O–H stretching vibration | Alginate, PEG, Carboxymethyl chitosan | Indicates presence of hydroxyl groups; confirms hydrophilic nature and hydrogen bonding capability of polymer matrix |
| 1586 cm⁻¹ | Asymmetric stretching of –COO⁻ groups | Alginate, Carboxymethyl chitosan, Potassium oxalate | Confirms presence of carboxylate groups and oxalate ions within formulation |
| 1404 cm⁻¹ | Symmetric stretching of –COO⁻ groups | Alginate, Carboxymethyl chitosan, Potassium oxalate | Supports integrity of polymer backbone and oxalate incorporation |
| 1306 cm⁻¹ | Symmetric O–C–O stretching vibration | Potassium oxalate | Characteristic oxalate ion vibration indicating successful active ingredient incorporation |
| 1101 cm⁻¹ | C–O–C stretching vibration (glycosidic linkages) | PEG, Alginate, Carboxymethyl chitosan | Confirms presence of polysaccharide backbone and PEG structure |
| 1025 cm⁻¹ | C–O stretching vibration | PEG, Alginate, Carboxymethyl chitosan | Characteristic polysaccharide ether linkage; supports structural integrity of polymer matrix |

**Table 1: FTIR peak assignments of the potassium oxalate–based strip** **indicating functional groups and corresponding formulation components.**
